# Biological aging markers in South African women with HIV and variable exposure to childhood maltreatment

**DOI:** 10.64898/2026.10.15.26365601

**Published:** 2026-10-16

**Authors:** Kabelo Mabokane Simon Maloka, Georgina Spies, Stefan du Plessis, Sian Megan Joanna Hemmings, Scott Letendre, Soraya Seedat, Jacqueline Samantha Womersley

## Abstract

**Introduction:**

HIV and childhood maltreatment (CM) are both chronic stressors that disproportionately affect women, with each contributing to accelerated biological aging. Whether biological aging markers are convergent and associate with HIV and CM is unclear.

**Methods:**

We studied 238 women (122 with HIV), characterised for CM severity, and assessed biological aging using absolute telomere length (aTL), mitochondrial DNA copy number (mtDNAcn), magnetic resonance imaging-based brain-predicted age difference (MRI-BPAD), and DNA methylation age (DNAmAge) clocks. CM was measured with the Childhood Trauma Questionnaire–Short Form total score. We examined intercorrelations among biological aging markers to assess convergence and conducted linear regression models to test moderation by HIV status and CM severity.

**Results:**

PWH had shorter aTL, lower MRI-BPAD, and higher Horvath age acceleration (HorvathAA), HannumAA, PhenoAA, and extrinsic epigenetic age acceleration (EEAA). CM had limited associations with biological aging markers. Absolute TL correlated weakly with mtDNAcn ( *ρ* = 0.36), PhenoAA ( *ρ* = -0.18), and EEAA ( *ρ* = -0.18), while MRI-BPAD correlated weakly with PhenoAA ( *r* = 0.20). DNAmAge clocks were moderately to strongly intercorrelated ( *r* = 0.35 – 0.93). No robust two- or three-way moderation effects were observed after false discovery rate correction.

**Conclusion:**

Our findings support a multidimensional pattern of biological aging, with substantial convergence among epigenetic aging measures but limited relationships across different markers, largely independent of HIV status and CM severity. Longitudinal approaches are needed to clarify how distinct biological aging markers relate to one another and to health outcomes over time in vulnerable populations.

